# Monitoring the Well-being of Older People by Energy Usage Patterns: Systematic Review of the Literature and Evidence Synthesis

**DOI:** 10.2196/41187

**Published:** 2023-03-31

**Authors:** Sophie A Korenhof, Yuan Fang, Jie Luo, Tischa J M van der Cammen, Hein Raat, Amy van Grieken

**Affiliations:** 1 Department of Public Health Erasmus University Medical Center Rotterdam Netherlands; 2 Section of Geriatrics Department of Internal Medicine Erasmus University Medical Center Rotterdam Netherlands; 3 Department Human-Centered Design Faculty of Industrial Design Engineering Delft University of Technology Delft Netherlands

**Keywords:** smart energy meter, healthy aging, activities of daily living, independent living, monitoring, older adults, devices, risk, well-being, effectiveness, design, safety

## Abstract

**Background:**

Due to the aging population, there is a need for monitoring well-being and safety while living independently. A low-intrusive monitoring system is based on a person’s use of energy or water.

**Objective:**

The study's objective was to provide a systematic overview of studies that monitor the health and well-being of older people using energy (eg, electricity and gas) and water usage data and study the outcomes on health and well-being.

**Methods:**

CENTRAL, Embase, MEDLINE (Ovid), Scopus, Web of Science, and Google Scholar were searched systematically from inception until November 8, 2021. The inclusion criteria were that the study had to be published in English, have full-text availability, target independent-living people aged 60 years and older from the general population, have an observational design, and assess the outcomes of a monitoring system based on energy (ie, electricity, gas, or water) usage on well-being and safety. The quality of the studies was assessed by the QualSyst systematic review tool.

**Results:**

The search strategy identified 2920 articles. The majority of studies focused on the technical algorithms underlying energy usage data and related sensors. One study was included in this review. This study reported that the smart energy meter data monitoring system was considered unobtrusive and was well accepted by the older people and professionals involved. Energy usage in a household acted as a unique signature and therefore provided useful insight into well-being and safety. This study lacked statistical power due to the small number of participants and the low number of observed events. In addition, the quality of the study was rated as low.

**Conclusions:**

This review identified only 1 study that evaluated the impact of an energy usage monitoring system on the well-being and safety of older people. The absence of reliable evidence impedes any definitive guidance or recommendations for practice. Because this emerging field has not yet been studied thoroughly, many questions remain open for further research. Future studies should focus on the further development of a monitoring system and the evaluation of the implementation and outcomes of these systems.

**Trial Registration:**

PROSPERO CRD42022245713; https://www.crd.york.ac.uk/prospero/display_record.php?RecordID=245713

## Introduction

The number of older people is increasing worldwide due to a global increase in life expectancy [1]. Between 2015 and 2050, the percentage of “older people,” in this study defined as those 65 years and older, will nearly double as a proportion of the global population, from 12% to 22%, respectively [2]. As a result, the old-age dependency ratio (ie, the ratio of people aged 20-64 years to people 65 years and older) has declined from around 4 working adults for every person older than 65 years in 2001 to fewer than 3 in 2020 [3]. This means that roughly 1 adult older than 65 years is financially dependent on less than 3 working adults (aged 20-64 years) at present [4]. In addition, the proportion of single-person households in the European Union increased as well, from 25% in 2010 to 35% in 2017 [5]. Meanwhile, more and more older people live independently—the “aging in place” trend [6,7]. Independent living means noninstitutionalized living, with the possibility of extra professional help if needed [8].

Technologies are being developed within the health care sector to enable older people to live independently for longer. There is a wide variety of health technologies available to monitor symptoms of chronic disease; in the World Health Organization report on digital health, a complete overview is provided [9]. Technologies include the Internet of Things, virtual care, remote monitoring, artificial intelligence, big data analytics, blockchain, and smart wearables, but also platforms and tools enabling data exchange and storage and tools enabling remote data capture [9]. These technologies aim to provide the necessary tools to monitor the symptoms of chronic conditions like dementia. Research by Brownsell et al [10] showed that an individual’s health status can be determined based on simple interactions between an individual and their immediate environment. This determination is the premise of activities of daily living (ADL) monitoring technologies, which aim to support the safety of the older person and enable both formal and informal caregivers to check in with the older person. For example, sensors are placed inside the homes of older people to detect movement, sleep patterns, occupancy, living conditions, or appliance usage [10]. There is a vast amount of literature available describing the potential of smart homes [10-12]. For example, a longitudinal study by Kaye et al [12] showed that the placement of infrared motion sensors could monitor walking speed and other in-home activity metrics. These solutions, however, require the installation and maintenance of specific hardware and software in the house or apartment of the older person. Additional sensors in the house or apartment, such as cameras, motion detectors, or heart rate monitors, can also be considered obtrusive, expensive, and violate the resident’s privacy [11,13].

A potential low-intrusive system for monitoring may be designed using smart energy meter data. Smart energy meters are increasingly present in houses and apartments worldwide, automatically measuring and recording energy usage. A smart energy meter is a meter that keeps track of the energy supply and sends meter readings to the energy supplier automatically [14]. A study by Berg Insight [15] reported that the expectation is to reach 72% of smart electricity meter coverage in Europe by 2026. In 2024, in the EU, more than 100 million smart meters for electricity and over 50 million for gas will be rolled out [16]. This increase in coverage provides an opportunity for this type of research. The growing availability of smart energy meters and the relative simplicity of recording, storing, and transmitting data have created a potential opportunity to monitor activity among older people to support well-being and safety.

Specifically, age-related diseases, such as cognitive decline, have a direct impact on ADLs [17,18]. Based on the disaggregation of energy usage, ADL patterns can be established in a simple, unobtrusive, and inexpensive way [19]. By disaggregating the total energy load, it is possible to determine which appliances are being used on a certain day [20-23]. This technique, also known as nonintrusive load monitoring, makes it possible, by using algorithms, to infer the fine-grained energy usage patterns of different appliances in the household [20,24-26]. This energy usage pattern could be linked to health-related activities, such as cooking, which can therefore be used as a proxy for the general health and safety of the persons living in this household and possibly anticipate accidents or hospitalization [27].

The aim of this review was 2-fold. The first aim was to provide an overview of existing evidence describing initiatives that developed a monitoring system for the well-being and safety of independent-living older people using energy (ie, electricity and gas) and water usage data. For the remainder of the paper, we will use “energy” to refer to electricity, gas, and water. The second was to provide an overview of the outcomes of these systems on the well-being of older people. Moreover, if implementation outcomes were described, these were also reported.

## Methods

### Search Strategy

This systematic review was registered at PROSPERO (ID 245713). The PRISMA (Preferred Reporting Items for Systematic Reviews and Meta-Analyses) statement was used as a guideline (Multimedia Appendix 1) [28]. A systematic literature search was conducted within the databases CENTRAL, Embase, MEDLINE (Ovid), Scopus, Web of Science, and Google Scholar in November 2021 to identify relevant studies. The following keywords were included in the search: “smart meter,” “energy meter,” “electric meter,” “gas meter,” “water meter,” “independent living,” “monitoring,” “support,” “activity recognize,” “anomaly,” “daily life activity,” and “community-dwelling.” The search strategy was determined in collaboration with a medical information specialist; the full search strategy can be found in Multimedia Appendix 2. Additional articles were added via a manual search for eligible articles based on the reference lists of the included articles.

### Inclusion and Exclusion Criteria

#### Language

The inclusion criteria included studies published in English. Non-English studies were excluded from the study.

#### Article Availability

Only full articles were included. Conference abstracts and proceedings were excluded; for conference abstracts, corresponding authors were contacted for full text; these articles were excluded if they were unavailable.

#### Target Population

The study is based on independent-living people 60 years and older from the general population. Studies recruiting only clinical populations (eg, patients diagnosed with dementia) and studies among nonhumans were excluded.

#### Type of Study

Studies with an observational design (ie, a design where the participant is observed and analyzed in their natural or real-world setting) and that assessed the outcomes of a monitoring system based on energy usage on outcomes regarding the well-being and safety of the older people were included. Studies assessing only feasibility were excluded.

#### Study Focus

Studies that evaluated the monitoring system based on energy usage, including electricity, gas, or water were included. Studies using only wearable appliances to determine the well-being of the study participants (eg, heart rate appliances and movement sensors) were excluded.

### Study Selection

All references were exported and managed using EndNote (version X9; Clarivate Analytics). After duplicate records were removed, title and abstract screening were performed independently by 2 reviewers (SK and YF), based on the predetermined inclusion and exclusion criteria described above. Relevant articles were retrieved for full-text reading and further review by 2 reviewers (SAK and YF). The 2 reviewers discussed disagreements until they agreed. The remaining disagreements were discussed with a third author (AvG) until a consensus was reached.

### Data Extraction

A predetermined data extraction form was filled in with the extracted data from the included studies. Extracted information included the first author, year of publication, country, population and characteristics (ie, number of participants, type of population, and age of population), study design, study period, type of monitoring system, health-related outcomes, and effect size. This process was conducted by 2 researchers (SK and YF) independently. Discrepancies were resolved through discussion.

### Quality Appraisal

The quality of the included study was assessed with the QualSyst (standard quality assessment criteria) systematic review tool of Kmet et al [29]. This checklist contains 10 items, which were scored from 0 points (no), 1 point (partial), to 2 points (yes). All scores were summed and consequently divided by the total possible sum score to calculate the quality score per study. With this final score, the quality of the study was then rated as high (≥0.75), medium (≥0.55 and < 0.75), or low (<0.55) [29]. This process was performed independently by 2 researchers (SAK and YF). Discrepancies between the researchers were discussed until a consensus was reached.

## Results

### Search Results

**Figure 1:**
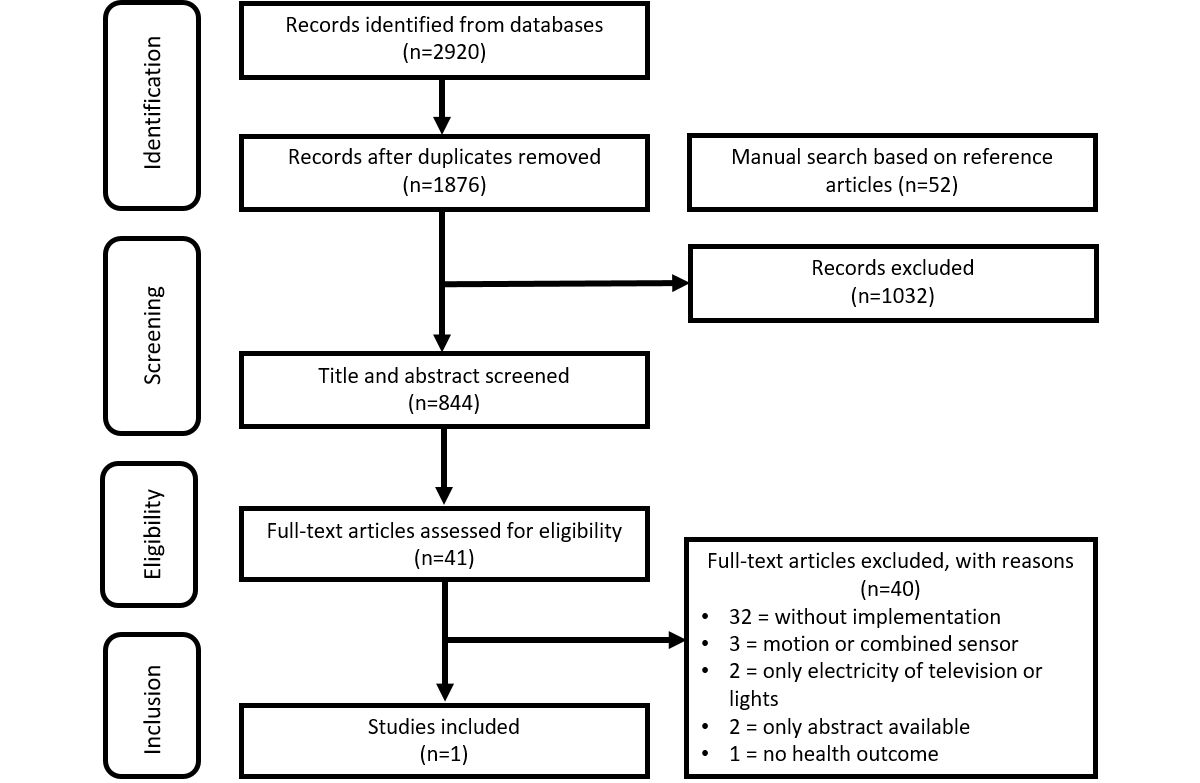
PRISMA (Preferred Reporting Items for Systematic Reviews and Meta-Analyses) flow diagram.

The search strategy identified 2920 articles in the selected databases. After deduplication, 1876 articles remained for the title and abstract screening. Based on the reference lists of the included articles and screened on title and abstract, 52 articles were added via a manual search for eligible articles. After title and abstract screening, 844 articles remained. After screening the remaining full-text articles for eligibility, 41 articles were eligible for inclusion in this systematic review. Finally, 1 article was included; in this article, 2 substudies were reported [27]. The PRISMA flow diagram is presented in Figure 1. The characteristics of the included study are described in Table 1.

**Table 1:** Characteristics of the included study.

First author (year of publication)	Country	Participants included, n			Study period			Type of study population		Study design			Age at participation (years)			Determinants or outcome		Level of quality
		Study 1	Study 2	Study 1		Study 2			Study 1		Study 2	Study 1		Study 2				
Noury et al [27] (2011)	France	13	12	Before 2008		2008	Community-dwelling older citizens (1-person household)		Single-arm trial		Single-arm trial	Unknown		80.5	Detection of ADL^a^, validity of energy use (daily index of activity), acceptability of the monitoring system		Low^b^	

^a^ADL: activities of daily living.

^b^Low quality as rated with the QualSyst systematic review tool by Kmet et al [29].

### Study Characteristics and Results

We included 1 article that included the report of 2 substudies. In this study, Noury et al [27] aimed to develop a system for the remote monitoring of large populations of older people living independently at home. In addition, they developed an ADL index and evaluated the relevance and acceptability of the monitoring system as a whole. They used electricity data to study ADLs. They built a unique referential (ie, individual energy use pattern) for each subject, constructed from the mean energy usage of the selected ADLs: food preparation and eating, hygiene, and turning off appliances during diurnal and nocturnal periods. A unique ADL index was computed for each ADL and compared to the corresponding mean value in the referential. The data was obtained by a detector on the residential power line to monitor the energy usage of each electrical appliance in a home [30]. This electricity usage monitoring system could memorize a “signature” for each appliance during a learning phase. Noury et al [27] referred to both ADLs and instrumental ADLs (IADLs). IADLs include housekeeping tasks such as “turning off appliances.” For the remainder of the paper, we will use ADLs to refer to both ADLs and IADLs [31].

The study consisted of 2 experiments, both conducted with older people living in single-person households [27]. The first experiment was conducted in 2005 with 13 participants from the LI2G lab (Laboratoire Interuniversitaire de Gérontologie de Grenoble) in Grenoble, France. The second experiment was conducted with 12 participants, aged 80.5 (SD 3.2) years, in Sable-sur-Sarthe and Chatillon, France, in 2008. In this study, 3 ADLs (food preparation and eating, hygiene, and turning off appliances) in 4 time periods (morning, afternoon, evening, and night) were studied. Two additional activity levels (diurnal and nocturnal) were also studied, and an ADL index was built, which was validated by a social worker involved with the participants. The results of the study showed that electricity usage data could be a useful method to monitor independent-living older people’s ADLs. In addition, they tested this in 2 separate experiments in the population and showed that ADLs can be detected through electricity usage monitoring. They also showed that deviations from the normal 24-hour pattern could be linked to older people’s ADL patterns, which were subsequently linked with their health status and interpreted by a social worker. Furthermore, the study was well-accepted by both the participants and the social workers involved, and it was considered a little intrusive. However, participants were afraid of the costs involved in data transfer communications.

### Quality Appraisal

The quality of the study, measured with the QualSyst systematic review tool of Kmet et al [29], was rated as low (<0.55). Overall, the QualSyst systematic review tool rated the quality of the evidence in this study as low, as it only included a small number of participants (n=25), and the objective, connection to the theory, data collection, data analysis, and outcomes were incompletely reported. Furthermore, no information on sampling strategy, verification, or reflexivity was reported. Moreover, the conclusion was only partially supported by the results. Next to that, participants self-reported their ADLs, and the questionnaires were not well described [27].

## Discussion

### Principal Findings

With this systematic review, we aimed to provide an overview of existing initiatives using energy data to monitor the well-being and safety of older people and to describe the outcomes of these energy data monitoring systems. Only 2 substudies were identified in the literature and reported in 1 article [27]. This 1 article was rated as low quality. Moreover, the outcomes for the well-being of older people were not visible. However, it did indicate that independent-living older people and social workers who participated in the study considered the energy usage monitoring system to be a little intrusive and a reliable and potentially beneficial solution for the time management of professionals involved in older persons’ care.

### Implementation of Monitoring Energy Usage to Support Independent Living

**Figure 2:**
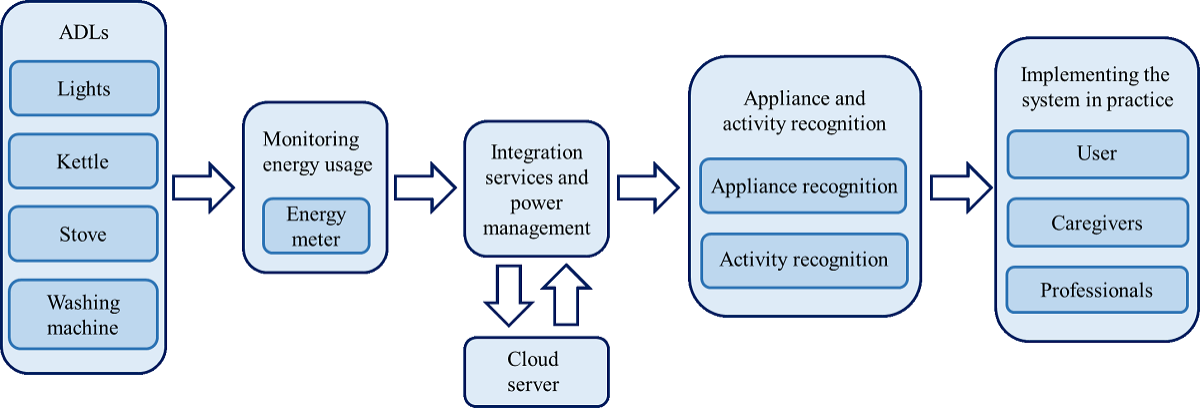
Energy usage monitoring elements. ADL: activities of daily living.

Monitoring energy usage to support independent living requires a process of step-by-step evaluation and testing. It can be described as consisting of 3 elements: monitoring energy usage, appliance and activity recognition, and implementing the system in practice. Figure 2 illustrates the elements that are involved in studying the feasibility of energy usage monitoring.

First, regarding the ways of monitoring energy usage, there are 2 main ways of data collection: overall data from all appliances, via, for example, the smart energy meter, or data from just a single appliance. Both approaches have their advantages. Data from a single appliance does not need to be disaggregated; it is already known where the energy usage comes from. For data from the smart electricity meter, however, all electrical activity within the household is included, which requires disaggregation. The Noury et al [27] study described placing a detector on the residential power line, placed inside the main electrical supply cabinet, which monitored electricity usage and acted as a sensor for the total household consumption.

Second, regarding appliance and activity recognition, energy usage monitoring enables the identification of appliances through their energy usage patterns [17]. Subsequently, this pattern can then be used to identify ADL patterns. One way is appliance detection, whereby a pattern of a specific appliance can be recognized within the total energy usage; another way is historical ADL pattern recognition, whereby current energy usage is compared to historical energy usage [17]. Potentially, problems that have a rising prevalence with age, such as insomnia and possibly cognitive decline, can cause problems in ADLs [27]. Energy usage monitoring may, for example, detect deviations from the “normal” 24-hour pattern such as activity during the night [27].

The next step in appliance and activity recognition would be verifying the algorithms with real-life data. The studies identified in this review focused on testing algorithms mainly on already existing energy data or computer-generated energy data. These studies reported a reasonable (50%) to good (84%) accuracy for recognizing (kitchen) appliances [21,32-34].

### Comparison to Prior Work

However, few studies were conducted to test the accuracy and outcomes of an energy usage monitoring system in real-life settings [17]. Billis et al [35] used smart television data to extract meaningful information about television usage patterns and subsequently associate them with the clinical findings of experts. Another study by Franco et al [36] tested the feasibility of a system for noninvasive monitoring of subjects at home by recording electrical activity from room lighting and other electrical domestic appliances. This type of monitoring—monitoring by specific types of appliances—is less sensitive to deviations from the average 24-hour energy usage pattern compared to an energy usage monitoring system operating via the smart meter because these particular appliances do not include the total activity of a household. However, the advantage of this system is that the collected data does not need to be disaggregated; it is immediately clear which appliance was used [35,36].

Lastly, it is needed to implement the system in practice and collect information on the impact on well-being, safety, and usability. The only article included in this review, by Noury et al [27], included energy usage monitoring, appliance and activity recognition, as well as testing the system in practice. Although the article of Noury et al [27] concluded that such a system is promising and appreciated by users, more research is needed to further confirm and elaborate on these findings. Moreover, to implement a monitoring system in practice, studies are needed to evaluate the design and usability of the system, for example, the client interface and communication.

### Challenges to Implementing Energy Use Monitoring

Although energy usage monitoring is a promising method, some challenges should also be mentioned. We divided the challenges according to the levels of the socio-ecological framework [37]. First, on a personal level, energy usage monitoring is considered unobtrusive; however, privacy-related issues are still involved [32]. A study by Kolter and Johnson [32] reported that sharing real-time energy usage data can potentially be harmful since these data can easily be linked to being at home or not. During the rollout of smart energy meters on an international level, concern has been expressed within the population about the possibility of privacy breaches [38]. These discussions and issues should be considered in future studies evaluating the potential of smart-energy monitoring systems. Therefore, separating real-time energy usage data from identifiable personal data is essential. Furthermore, older people’s attitudes toward technology are relatively mixed [39]. Those who fear technology use are generally afraid of privacy breaches, loss of self-determination, and the replacement of human contact [39,40]. Others embrace technology and are more dependent on it, consequently more easily accepting possible negative side effects [39,40]. Counterintuitively, longer independent living can be supported by technologies and, therefore, might protect against a certain loss of privacy that will be in place when living in an institution [41].

Second, on a community and structural level, data collection from a smart energy meter can be easily achieved with a reliable internet connection, yet even in Europe, not everyone has access to the internet [42]. In addition, ADL patterns in older people are not always stable. It is relatively easy to detect deviations in ADL patterns; however, a deviation is not necessarily related to a change in health and could also mean that this person does not have a regular lifestyle [10]. Therefore, it can be challenging to relate deviations from normal ADL patterns to changes in well-being [10]. Discovering deviations in the lifestyle or ADL pattern would require longer-term monitoring. A potential advantage of monitoring electricity consumption by smart meters is that smart meters can perform this sort of long-term monitoring.

### Strengths and Limitations

To the best of our knowledge, this is the first systematic review addressing ways to monitor the well-being and safety of older people using energy use data and describing their outcomes. Furthermore, we used a broad search strategy, including electricity, gas, and water, to identify all potential studies on this topic.

First, the main limitation of this systematic review is the lack of studies that could be included after screening—only 1. Overall, the QualSyst systematic review tool rated the quality of evidence in this study as low, as it only included a small number of participants (n=25), and the objective, connection to the theory, data collection, data analysis, and outcomes were incompletely reported. Furthermore, no information on sampling strategy, verification, or reflexivity was reported. Moreover, the conclusion was only partially supported by the results. Next to that, participants self-reported their ADLs, and the questionnaires were not well-described [27]. The lack of studies might have induced selection bias. Second, another methodological consideration of this review is that publication bias cannot be ruled out as only peer-reviewed articles in the English language were included. Third, this review studied older people 60 years and older; there is no set age limit to define “older” [2,43]. In general, with age and decreasing mobility, the chances of unsafe situations increase. For example, adults older than 60 years endure the greatest number of fatal falls [44]. Nevertheless, implementing an energy-based monitoring system may also be useful for other subgroups, independent of age. If an age limit had been set, the conclusions of the review could have better suited a defined age group; now, they are better suited for various subgroups. Fourth, there are several ways, technically, to monitor energy data. However, in this review, we aimed to evaluate the impact of energy monitoring on the well-being and safety of independent-living older people. So, we have limited ourselves to the broad outlines of all technical energy activity monitoring possibilities.

### Future Directions

The findings from this study indicate that energy usage monitoring may have the potential to aid in monitoring independent-living older people. Currently, the care for older people is organized more and more at home in Western countries [7]. Consequently, there will be relatively less capacity within high-need facilities for the rising numbers of older people [7].

First, further investigation of energy usage monitoring and the recognition of appliances and activities is required. Specifically, research is needed to examine whether, how, and for which subpopulations energy usage monitoring has potential. Heterogeneity between people increases with age, with an observed peak at 70 years; independent-living older people are therefore not a homogeneous group [45]. Furthermore, there are many levels of activity within a population, making it harder to build one system for the general population [46]. In line herewith, investigating the combination of electricity usage monitoring with other types of monitoring, such as via smart gas or water meters, could have great potential for increasing accuracy [47], especially as not all ADLs can be monitored by electricity usage, such as using the toilet or getting dressed. In addition, not all household appliances can be detected reliably yet [24]. Currently, smart electricity meters are the most installed; smart gas and water meters are less common but are also projected to increase shortly [15,48]. Household water usage can be disaggregated in various ways to produce similar data to electricity usage [49,50]. Smart water meters are at present mostly used for leakage detection and diminishing water use, but this information could also be used for ADL detection [51].

Second, it is recommended to implement the system in practice. Moreover, it is recommended that future studies apply a longitudinal study design with a larger sample size and longer study duration. Noury et al [27] included only 25 participants, who were followed up for 6 months. Since energy usage patterns are unique, it is important to include enough participants to have a representative sample of the target population [21]. Energy usage monitoring relies on energy pattern detection. Since energy usage is highly dependent on external factors like weather conditions, temperature, and the available appliances in a household, it is useful to follow a household for a longer period to take these factors into account and detect changes in 24-hour patterns.

Studies will also benefit from better measurement of well-being and safety outcomes. Since it is an emerging field, there have not been many scientific trials conducted in this field yet. The well-being and safety outcomes of energy usage monitoring and setting up a monitoring system based on this have not been well described yet. Noury et al [27] do mention health outcomes; however, it was not described which specific health outcomes were examined or how these health outcomes were measured. The other screened articles have not mentioned health outcomes at all.

### Conclusions

In this systematic review, we aimed to provide an overview of existing evidence describing initiatives that developed and tested the outcomes of a system that monitors the well-being and safety of independent-living older people using energy usage data. Although only 1 article was included, which described 2 substudies that did not have sufficient power for definitive guidance for research and practice, this review has provided an overview of the current literature on energy usage monitoring systems for the well-being and safety of independent-living older people. The absence of reliable evidence impedes any definitive guidance or recommendations for practice. Future studies are recommended to further gain insight into both the technical development of a smart-energy usage monitoring system as well as the implementation of a system and its outcomes for older people and their caregivers.

## Appendix

Multimedia Appendix 1 PRISMA (Preferred Reporting Items for Systematic Reviews and Meta-Analyses) statement.

Multimedia Appendix 2 Full search strategy.

## Abbreviations

ADL: activities of daily living
IADL: instrumental activities of daily living
PRISMA: Preferred Reporting Items for Systematic Reviews and Meta-Analyses
QualSyst: standard quality assessment criteria

## Data Availability

Data sharing is not applicable to this study as no data sets were generated or analyzed during this study.
