# Supplementary material for: Monitoring the Well-being of Older People by Energy Usage Patterns: Systematic Review of the Literature and Evidence Synthesis
Source: JMIR Aging. 2023 Mar 31;6:e41187. doi: 10.2196/41187 (PMC10131843; doi:10.2196/41187)
Supplement: Multimedia Appendix 2 [file aging_v6i1e41187_app2.pdf]

Multimedia appendix 2: Full search strategy

Embase

(((electric* OR energy OR gas OR water) NEXT/1 (meter*)) OR ((smart OR wise) NEXT/1 (meter OR home*)) OR smarthouse OR smartmeter OR (sensor* NEAR/3 (enrich* OR rich*) NEAR/3 (home* OR environment*))) AND ('independent living'/de OR 'monitoring'/de OR 'health status'/de OR 'safety'/de OR 'home safety'/de OR 'community dwelling person'/de OR 'falling'/de OR 'daily life activity'/exp OR 'ambulatory monitoring'/de OR 'automated pattern recognition'/de OR sanitation/de OR 'personal hygiene'/de OR 'home monitoring'/de OR telehealth/de OR telemedicine/de OR 'human activities'/de OR 'data mining'/de OR algorithm/de OR 'wellbeing'/exp OR 'quality of life'/exp OR 'quality of life assessment'/exp OR (anomal* OR pattern* OR activit* OR recogni* OR monitoring OR (independent* NEAR/3 living) OR ((changes OR status) NEAR/3 health) or safely OR safety OR data-mining OR big-data OR community-dwell* OR Aging-in-Place OR living-alone OR fall OR falls OR accident* OR support* OR (daily NEAR/6 (life OR living) NEAR/6 activit*) OR sanitation OR (personal* NEAR/3 hygien*) OR telehealth OR tele-health OR telemedicine OR tele-medicine OR (human NEAR/3 activit*) OR algorithm* OR self-sufficien* OR wellbeing OR well-being OR (quality NEAR/3 life) OR qol OR hrqol OR hrql):ab,ti) NOT ([Conference Abstract]/lim)

26-2-2021: 815 articles with ‘advanced search’

Medline ovid

(((electric* OR energy OR gas OR water) ADJ (meter*)) OR ((smart OR wise) ADJ (meter OR home*)) OR smarthouse OR smartmeter OR (sensor* ADJ3 (enrich* OR rich*) ADJ3 (home* OR environment*))) AND ((anomal* OR pattern* OR activit* OR recogni* OR monitoring OR (independent* ADJ3 living) OR ((changes OR status) ADJ3 health) or safely OR safety OR data-mining OR big-data OR community-dwell* OR Aging-in-Place OR living-alone OR fall OR falls OR accident* OR support* OR (daily ADJ6 (life OR living) ADJ6 activit*) OR sanitation OR (personal* ADJ3 hygien*) OR telehealth OR tele-health OR telemedicine OR tele-medicine OR (human ADJ3 activit*) OR algorithm* OR self-sufficien* OR wellbeing OR well-being OR (quality ADJ3 life) OR qol OR hrqol OR hrql).ab,ti.)

24-2-2021: 361 articles https://ovidsp.dc2.ovid.com/ovid-b/ovidweb.cgi#

26-2-2021: 452 articles https://ovidsp.dc1.ovid.com/ovid-b/ovidweb.cgi

03-03-2021: 660 articles (Medline (ALL)) https://ovidsp.dc2.ovid.com/ovid-b/ovidweb.cgi

Web of science

TS=((((electric* OR energy OR gas OR water) NEAR/1 (meter*)) OR ((smart OR wise) NEAR/1 (meter OR home*)) OR smarthouse OR smartmeter OR (sensor* NEAR/2 (enrich* OR rich*) NEAR/2 (home* OR environment*))) AND (((anomal* OR pattern* OR activit* OR recogni* OR monitoring OR safely OR safety OR data-mining OR big-data OR accident* OR algorithm* OR support*) AND (elderly OR handicap* OR older OR aged)) OR (independent* NEAR/2 living) OR ((changes OR status) NEAR/2 health) OR community-dwell* OR Aging-in-Place OR living-alone OR fall OR falls OR (daily NEAR/5 (life OR living) NEAR/5 activit*) OR sanitation OR (personal* NEAR/2 hygien*) OR telehealth OR tele-health OR telemedicine OR tele-medicine OR (human NEAR/2 activit*) OR self-sufficien* OR wellbeing OR well-being OR (quality NEAR/2 life) OR qol OR hrqol OR hrql)) AND DT=(article)

24-2-2021: 1018 articles
https://apps.webofknowledge.com/summary.do?product=WOS&doc=1&qid=2&SID=D6G1GvZsNJLT7rJpnmJ&search_mode=AdvancedSearch&update_back2search_link_param=yes

03-03-2021: 1021 articles

https://apps.webofknowledge.com/summary.do?product=WOS&parentProduct=WOS&search_mode=AdvancedSearch&parentQid=&qid=1&SID=F2GNQCWT7LJeeuwMLHi&&update_back2search_link_param=yes&page=21

Scopus

TITLE-ABS-KEY((((electric* OR energy OR gas OR water) W/1 (meter*)) OR ((smart OR wise) W/1 (meter OR home*)) OR smarthouse OR smartmeter OR (sensor* W/2 (enrich* OR rich*) W/2 (home* OR environment*))) AND (((anomal* OR pattern* OR activit* OR recogni* OR monitoring OR safely OR safety OR data-mining OR big-data OR accident* OR algorithm* OR support*) AND (elderly OR handicap* OR older OR aged)) OR (independent* W/2 living) OR ((changes OR status) W/2 health) OR community-dwell* OR Aging-in-Place OR living-alone OR fall OR falls OR (daily W/5 (life OR living) W/5 activit*) OR sanitation OR (personal* W/2 hygien*) OR telehealth OR tele-health OR telemedicine OR tele-medicine OR (human W/2 activit*) OR self-sufficien* OR wellbeing OR well-being OR (quality W/2 life) OR qol OR hrqol OR hrql)) AND DocType(Ar)

24-2-2021: 1197 articles

https://www.scopus.com/results/results.uri?sort=plf-f&src=s&sid=a49b209fad3d424b0667e29556970251&sot=a&sdt=a&sl=818&s=TITLE-ABS-KEY%28%28%28%28electric*+OR+energy+OR+gas+OR+water%29+W%2f1+%28meter*%29%29+OR+%28%28smart+OR+wise%29+W%2f1+%28meter+OR+home*%29%29+OR+smarthouse+OR+smartmeter+OR+%28sensor*+W%2f2+%28enrich*+OR+rich*%29+W%2f2+%28home*+OR+environment*%29%29%29+AND+%28%28%28anomal*+OR+pattern*+OR+activit*+O

R+recogni*+OR+monitoring+OR+safely+OR+safety+OR+data-mining+OR+big-data+OR+accident*+OR+algorithm*+OR+support*%29+AND+%28elderly+OR+handicap*+OR+older+OR+aged%29%29+OR+%28independent*+W%2f2+living%29+OR+%28%28changes+OR+status+%29+W%2f2+health%29+OR+community-dwell*+OR+Aging-in-Place+OR+living-alone+OR+fall+OR+falls+OR+%28daily+W%2f5+%28life+OR+living%29+W%2f5+activit*%29+OR+sanitation+OR+%28personal*+W%2f2+hygien*%29+OR+telehealth+OR+tele-health+OR+telemedicine+OR+tele-medicine+OR+%28human+W%2f2+activit*%29+OR+self-sufficien*+OR+wellbeing+OR+well-being+OR+%28quality+W%2f2+life%29+OR+qol+OR+hrqol+OR+hrql%29%29+AND+DocType%28Ar%29&origin=searchadvanced&editSaveSearch=&txGid=d4ae718fb17a49c89b8284443fde0ed1

03-03-2021: 1203 articles

https://www.scopus.com/results/results.uri?sort=plf-f&src=s&sid=354ae88727221b35d313e517f948033c&sot=a&sdt=a&sl=818&s=TITLE-ABS-KEY%28%28%28%28electric*+OR+energy+OR+gas+OR+water%29+W%2f1+%28meter*%29%29+OR+%28%28smart+OR+wise%29+W%2f1+%28meter+OR+home*%29%29+OR+smarthouse+OR+smartmeter+OR+%28sensor*+W%2f2+%28enrich*+OR+rich*%29+W%2f2+%28home*+OR+environment*%29%29%29+AND+%28%28%28anomal*+OR+pattern*+OR+activit*+OR+recogni*+OR+monitoring+OR+safely+OR+safety+OR+data-mining+OR+big-data+OR+accident*+OR+algorithm*+OR+support*%29+AND+%28elderly+OR+handicap*+OR+older+OR+aged%29%29+OR+%28independent*+W%2f2+living%29+OR+%28%28changes+OR+status+%29+W%2f2+health%29+OR+community-dwell*+OR+Aging-in-Place+OR+living-alone+OR+fall+OR+falls+OR+%28daily+W%2f5+%28life+OR+living%29+W%2f5+activit*%29+OR+sanitation+OR+%28personal*+W%2f2+hygien*%29+OR+telehealth+OR+tele-health+OR+telemedicine+OR+tele-medicine+OR+%28human+W%2f2+activit*%29+OR+self-sufficien*+OR+wellbeing+OR+well-being+OR+%28quality+W%2f2+life%29+OR+qol+OR+hrqol+OR+hrql%29%29+AND+DocType%28Ar%29&origin=searchadvanced&editSaveSearch=&txGid=4985255509f7b0f84199be2a093c2f08

Cochrane CENTRAL

(((electric* OR energy OR gas OR water) NEXT/1 (meter*)) OR ((smart OR wise) NEXT/1 (meter OR home*)) OR smarthouse OR smartmeter OR (sensor* NEAR/3 (enrich* OR rich*) NEAR/3 (home* OR environment*))) AND ((anomal* OR pattern* OR activit* OR recogni* OR monitoring OR (independent* NEAR/3 living) OR ((changes OR status) NEAR/3 health) or safely OR safety OR data next mining OR big next data OR community next dwell* OR Aging next in next Place OR living next alone OR fall OR falls OR accident* OR support* OR (daily NEAR/6 (life OR living) NEAR/6 activit*) OR sanitation OR (personal* NEAR/3 hygien*) OR

telehealth OR tele next health OR telemedicine OR tele next medicine OR (human NEAR/3 activit*) OR algorithm* OR self next sufficien* OR wellbeing OR well next being OR (quality NEAR/3 life) OR qol OR hrqol OR hrql):ab,ti)

24-2-2021: 18 articles (2 cochrane, 16 trials) https://www.cochranelibrary.com/advanced-search/search-manager

03-03-2021: 18 articles (2 cochrane, 16 trials) https://www.cochranelibrary.com/advanced-search/search-manager

Google scholar

"electricity|energy|gas|water meter"|"smart meter|home" "community-dwellers|dwelling"|"Aging-in-Place"|"living-alone|independently"|"independent living"

24-2-2021: 10.200 resultaten…
